# When left does not seem right: epigenetic and bioelectric differences between left- and right-sided breast cancer

**DOI:** 10.1186/s10020-022-00440-5

**Published:** 2022-02-05

**Authors:** Sofía Masuelli, Sebastián Real, Emanuel Campoy, María Teresita Branham, Diego Matías Marzese, Matthew Salomon, Gerardo De Blas, Rodolfo Arias, Michael Levin, María Roqué

**Affiliations:** 1 National Council of Scientific and Technological Research (IHEM-CONICET), PC:5500 Mendoza, Argentina; 2 Medical School, National University of Cuyo, PC:5500 Mendoza, Argentina; 3 Medical School, Mendoza University, PC:5500 Mendoza, Argentina; 4 Cancer Epigenetics Laboratory, Health Research Institute of the Balearic Islands (IdISBa), Palma, Spain; 5 Department of Medicine, Keck School of Medicine, University of Southern California, Los Angeles, CA 90007 USA; 6 Allen Discovery Center at Tufts University, Medford, MA 02115 USA; 7 Exact Science Faculty, National University of Cuyo, PC:5500 Mendoza, Argentina

**Keywords:** Asymmetry, Laterality, Left, Right, Breast cancer

## Abstract

**Background:**

During embryogenesis lateral symmetry is broken, giving rise to Left/Right (L/R) breast tissues with distinct identity. L/R-sided breast tumors exhibit consistently-biased incidence, gene expression, and DNA methylation. We postulate that a differential L/R tumor-microenvironment crosstalk generates different tumorigenesis mechanisms.

**Methods:**

We performed in-silico analyses on breast tumors of public datasets, developed xenografted tumors, and conditioned MDA-MB-231 cells with L/R mammary extracts.

**Results:**

We found L/R differential DNA methylation involved in embryogenic and neuron-like functions. Focusing on ion-channels, we discovered significant L/R epigenetic and bioelectric differences. Specifically, L-sided cells presented increased methylation of hyperpolarizing ion channel genes and increased Ca^2+^ concentration and depolarized membrane potential, compared to R-ones. Functional consequences were associated with increased proliferation in left tumors, assessed by KI67 expression and mitotic count.

**Conclusions:**

Our findings reveal considerable L/R asymmetry in cancer processes, and suggest specific L/R epigenetic and bioelectric differences as future targets for cancer therapeutic approaches in the breast and many other paired organs.

**Supplementary Information:**

The online version contains supplementary material available at 10.1186/s10020-022-00440-5.

## Background

Some organs, such as the heart or viscera are asymmetric: their structures to the left and right of the body mid-plane are consistently different in all normal individuals (Monsoro-Burq and Levin 2018). Most other tissues are often believed to be symmetrical. However, major knowledge gaps exist about the degree to which paired structures could exhibit not only the fluctuating asymmetry of developmental noise but consistently biased asymmetry that might impact structure and function. Despite the general assumption that mammary glands are mere copies of one another, each gland has its own identity and presents left–right (L/R) asymmetries. During embryogenesis of bilateral organisms, lateral symmetry is broken at very early stages in a programmed and consistent way (Vandenberg and Levin 2010; Levin et al. 2016; Ma 2013). The establishment of the L/R axis is the start of a regulated patterning, through which asymmetric sides arise at morphological, functional and molecular levels (Monsoro-Burq and Levin 2018; Sutherland and Ware 2009; McDowell et al. 2016). Alterations in laterality decisions during development give rise in humans not only to a reversed laterality (*situs inversus*) but also to an increased susceptibility to other diseases (Soofi et al. 2021). In particular, tumors in bilateral organs such as breast, colon, kidney or lung, show subtle but significant differences at morphological, genetic, molecular and incidence levels (Robichaux et al. 2014; Yang et al. 2018). Research data from our group and others (of diverse disciplines such as embryogenesis, development, molecular oncology, or cellular biology), have proposed that the asymmetric tumor microenvironment of bilateral organs could be part of the explanation for the L/R differences in cancer (Levin et al. 2016; Ma 2013; Robichaux et al. 2014; Campoy et al. 2016; Atiya et al. 2019; Yi et al. 2021).

Tumor cells sense the environment and fire in consequence internal signals. By this, the tumor transcriptome differs from the surrounding normal tissue and acquires specific features. The gene expression shift that the tumor applies to face the environmental challenges depends on the surrounding tissue signals, especially during the initial tumorigenesis stages. Thus, it can be said that the microenvironment contributes to the decision-making strategy of a tumor to reach the cancer hallmarks (Hanahan and Weinberg 2011). In this context, epigenetics and bioelectricity have a crucial role since both constitute vehicles by which external signals reach and modulate the transcriptome in an experience-dependent and dynamic way.

Epigenetic modifications highly influence the biology of cancer. A key feature of cancer cells is to respond rapidly to environmental challenges, and this is mainly attributed to the dynamic plasticity of the epigenetic mechanisms. Epigenetic regulators have both writing and erasing capacities, so are therefore able to maintain a flexible transcriptome which is crucial for tumor development and survival [reviewed in Dawson (2017)]. In addition, epigenomes are also defined as the bridges between the environment and the phenotype (or transcriptome) (Tammen et al. 2013). Being more dynamic and reversible than the genome, epigenomic variations can rapidly provoke a transcriptomic shift without changing the genomic sequence. DNA methylation, the most studied epigenetic modification, presents a specific signature associated with some cancer types, suggesting a distinct interplay between the tumor epigenome and the surrounding tissue. Based on this, specific aberrant DNA methylation patterns have been proposed as predictive and prognostic markers for several cancer types (Okugawa et al. 2015; D'Errico et al. 2020; Singh et al. 2019; Almeida et al. 2019). Specifically, in breast cancer, previous work of our group has identified that the DNA methylation profiles of tumor suppressor genes correlate with prognosis index (Marzese et al. 2010, 2012), with tumor subtypes (Branham et al. 2012), migration and metastasis capacity (Urrutia et al. 2015; Marzese et al. 2014), benign mammary lesions (Marzese et al. 2011), and, more relevant for the topic of this study, with the laterality of the tumors (Campoy et al. 2016). In summary, tumor epigenome is influenceable by the microenvironment and can be associated with differential tumor behaviors.

Bioelectric gradients are considered epigenetic mediators in a broad sense of the word, since they can modify the transcriptome following environmental signals (Cortés-Mendoza et al. 2013; Tseng and Levin 2012; Penas and Navarro 2018). The flow of ions (inside the tumor and between the tumor and the microenvironment) enables the transmission of membrane potential patterns, which are maintained as information for survival decisions in response to external challenges (Levin et al. 2019; Levin 2021). Like epigenetics, bioelectric control is reprogrammable, rapid and dynamic, and is driven by physiological states that are not 1:1 mapped to specific genes (Levin 2021). Bioelectric states are acquired by ion flux through channels and pumps in the membrane and are transmitted to neighboring cells via gap-junctions. The current flux produces changes in membrane potentials, which in turn generate downstream signaling to regulate different cellular processes, e.g. proliferation (Blackiston et al. 2009), migration, differentiation, or gene expression. Therefore, it is accepted that cells of the same tissue share similar bioelectric states, which is maintained as non-genetic information. This is also applicable to the L/R sides of bilaterian bodies, where metabolic (Onjiko et al. 2016), epigenetic, bioelectric, and gene-expression differences (Mittwoch 2008) have been reported. Just to highlight an example, L/R bioelectric differences have been observed in Xenopus and chicken embryos, finding consistent voltage and ion transporter asymmetries as early as the 2nd cell division (Levin et al. 2002). These bioelectric differences subsequently regulate asymmetric gene expression to control the sidedness of asymmetric organs and paired structures such as eyes and neural crest derivatives (Pai et al. 2012a).

Differences in L/R bilateral tumors have been reported for several cancer types, like breast (Kenney et al. 2004; Garfinkel et al. 1959), colon (Yang et al. 2018; Baran et al. 2018; Sponholz et al. 2021), kidney (Ni et al. 2021; Guo et al. 2019), brain (Połczyńska et al. 2021), ovary (He et al. 2021), and eye (Hussain 2021). Particularly in breast tumors, in addition to the largely known increased L-side incidence (Busk and Clemmensen 1947; Roychoudhuri et al. 2006), interesting differences in gene expression (Robichaux et al. 2014; Mittwoch 2008), gland microbiota (Klann et al. 2020), mitochondria distribution (Mishra and Chan 2014) and methylation profiles (Campoy et al. 2016) have been reported. It is reasonable to postulate that L/R adult glands conserve memories of their asymmetric embryogenic development, maintaining different L/R bioelectric patterns. These patterns are, in part, constituted by “attractors” (such as morphogens, neurotransmitters, small molecules) that trigger downstream different signaling pathways and change transcription regulation (Levin 2021).

In this work, we hypothesize that tumorigenic breast processes face environmental challenges that differ between L/R sides, establishing a side-dependent tumor-microenvironment crosstalk reflected in bioelectric and epigenetic differences. A serendipitous finding during our previous research gave rise to the present work, when we discovered that DNA methylation patterns of female patient’s breast tumors clustered in two groups based on whether they were located on the L or R gland (Campoy et al. 2016). This striking observation opened the questions of whether these L/R differences were reproducible in an in-silico, in-vivo and/or in-vitro model, and if functional differences were associated with these epigenetic profiles. In this work, we developed in-silico, in-vivo and in-vitro approaches to address these questions.

## Methods

### Collection of in-silico data from public datasets

For gene methylation analyses, *Illumina Infinium Human Methylation-450* information was obtained from breast cancer TCGA dataset, available in the public platform cBioportal for Cancer Genomics (https://www.cbioportal.org/, repository Firehose Legacy of the Broad Institute). The DNA methylation data is found in the repositories as beta values, which are continuous ratios between 0 and 1, indicating the intensities between methylated and unmethylated alleles (0 being unmethylated and 1 fully methylated). For laterality data, clinical datasets were also retrieved from the same platform. We used 782 primary breast tumors for which DNA methylation data of ~ 16,000 genes plus anatomical location (L/R gland) was available. After curating the information, we calculated the L/R DNA methylation mean for each genomic region and ranked their absolute differences (called from now on differential methylation, DM) (note: for all experiments, differences are calculated as *left minus right*; since data are beta values, the L–R differences are between 0 and 1).

To find the cellular and molecular functions in which the most differentially methylated genes were involved, we performed gene enrichment analyses with the public tools Metascape (https://metascape.org, RRID:SCR_016620) and EnrichR (https://maayanlab.cloud/Enrichr/enrich, RRID:SCR_001575). For Metascape tool, the enrichment analyses were set as: minimum overlap = 3 and p-value cutoff = 0.001, with Gene priorization by Evidence Countins (GPEC). To establish the potential functional consequences of the proximal (up to 2.5 kb up and downstream) as well as the distal (up to 1 Mb up and downstream) genomic context of the differential methylated CpGs, GREAT analyses were performed (Genomic Regions Enrichment of Annotations Tool v4.0.0) (McLean et al. 2010). We used the basal plus extension configuration as a background setup “whole genome”, as recently shown (Emran et al. 2017).

For gene expression analyses for selected genes of interest, *Illumina HiSeq 2000 RNA Sequencing platform* of the University of North Carolina was obtained from 1168 primary breast cancer TCGA dataset for DNMTs, 1095 for TETs and 1060 for KI67, available in the UCSC (University of California Santa Cruz) Xena Functional Genomics explorer (http://xena.ucsc.edu/, RRID:SCR_018938). The RNA-Seq data are shown in the dataset as normalized log2 (x + 1) values and indicate an estimated gene expression level.

### Xenografts generation

The highly immunosuppressed Nod Scid Gamma mice (NOD.Cg-PrkdcscidIl2rgtm1Wjl/SzJ, NSG) (RRID:IMSR_JAX:005557) were obtained from Jackson Laboratory and were housed in a pathogen-free condition throughout the experimental duration. All procedures were performed following the consideration of animal welfare and were approved by the Institutional Committee for Care and Procedures of Laboratory Animals (CICUAL in Spanish) of the National University of Cuyo, Mendoza, Argentina. To perform the xenograft experiment, 6-week-old female NSG (20 g) mice were anesthetized with isofluorane 4% in O_2_, and injected with 1 × 10^6^ MDA-MB-231 cells (suspended in physiologic solution) in the 4th L/R breast glands. Mice were closely monitored, and tumor size was measured weekly. Five weeks after cell inoculation, the mice were sacrificed in a CO_2_ camera, and tumors were excised. Part of the tumors was set apart and frozen at − 80 °C for further DNA and RNA extractions (labeled as passage 0). The remaining parts were reimplanted in small pieces in 3 NSG mice, maintaining laterality (labeled as passage 1). The complete procedures were repeated in 3 more NSG mice to generate tumors passage 2.

### Nucleic acid extraction

DNA was extracted from xenograft tumor tissues and from MDA-MB-231 cells, using PureLink® Genomic DNA Kits, Mammalian Tissue and Mouse/Rat Tail Lysate (Catalog Numbers K1820-02, Invitrogen), following manufacturer’s protocol. RNA was extracted from MDA-MB-231 cells using a Trizol based protocol (TRIzol® Reagent (Life technologies, Catalog Numbers 15596-026).

### DNA methylation analyses by MS-MLPA and RRBS

To assess the methylation status of 50 CpG sites located on 40 genes, the MS-MLPA kits ME001 and ME002 (Catalog Numbers ME001-025R, ME002-025R) were used. The MS-MLPA assays were performed basically according to manufacturer’s recommendations (MRC-Holland, Amsterdam, The Netherlands, www.mrc-holland.com) (Nygren et al. 2005), introducing subtle modifications (i.e., extended restriction enzyme incubation time, separated ligation and digestion steps), to avoid background signals (Marzese et al. 2010). The fluorescent-labeled PCR products were separated by capillary electrophoresis (3500 Genetic Analyzer for Fragment Analysis, Applied Biosystems) and analyzed by GeneMarker v1.75 software (RRID:SCR_015661). A cutoff of 8% fluorescence signal was established to consider the site significantly methylated.

To assess an extended methylation analysis involving most of the genome CpG sites, a reduced restricted bisulfite sequencing (RRBS) assay was performed with the technical and bioinformatic assessment of the Genomic Unit–Consortium CATG-National Institute of Agricultural Technology (INTA) in Buenos Aires, Argentina. For this, 3 matched left and right passage-1 xenografted tumors were selected. The experimental steps consisted on: preparation of the libraries with Diagenode’s Premium RRBS kit (Diagenode, Cat. No. C02030032), sodium bisulfite conversion of the DNA samples and PCR amplification and sequencing of the generated fragments on an Illumina NextSeq 550 equipment. Quality control of sequencing reads was performed using FastQC® (Babraham Bioinformatics®, RRID:SCR_014583). Adapter removal was done using Trim Galore® version 0.4.1 (Babraham Bioinformatics®, RRID:SCR_011847). Reads were then aligned to the reference genome GRCh38 using Bismark v0.22.1.® (Babraham Bioinformatics®, RRID:SCR_005604), followed by methylation calling using the corresponding bismark functionality. The comparison between the RRBS data sets was carried out using methylKit® (Bioconductor®, RRID:SCR_005177), with the GRCh38 refGene and CpG island annotation from UCSC (University of California Santa Cruz, RRID:SCR_006553) genome browser. Bioinformatic filters were applied on the raw results, to select only human sequences aligned with the human reference genome GRCh38, discarding possible mice genomic interference. After methylation calling, and difference calling with Bioconductor 3.9, L/R DM with more than 10% difference were found in 2219 sites. For enrichment analysis (by Metascape) we eliminated duplicated genes (with more than one methylated site per gene), leaving 1288 genes for further analyses (Additional file 1: Table S1).

### Cell culture

Human breast cancer cell line MDA-MB-231 (ATCC, RRID:CVCL_0062) was kindly provided by Dr. Matias Sanchez (IMBECU Institute, Mendoza, Argentina) and passages 20–30 were used for this work. The cells were routinely tested for mycoplasma contamination. In general, cells were cultured in DMEM medium (Gibco by Life Technologies, Grand Island, NY, USA, # 112800-058) supplemented with 10% fetal bovine serum (Internegocios S.A, Mercedes, BA, Argentina), 100 U/mL of penicillin and 100 μg/mL streptomycin (Gibco by Life Technologies, Grand Island, NY, USA, #1796440), at 37 °C in a humidified atmosphere containing 5% CO_2_. For the extract-conditioned cultures, fetal bovine serum was reduced to 1%.

### L/R extract preparation and conditioned cell culture

Healthy L/R breast glands were obtained from plastic surgeries, provided by Dr. Cataneo from the Clinic of Plastic Surgery of Mendoza, after patients signed an informed consent previously approved by the Ethics Committee of the Medical School of the National University of Cuyo. Tissues were first disaggregated with a scalpel and the pieces were suspended in 25 mL of DMEM medium with Penicillin/Streptomycin 1% and incubated in a shaker for 24 h at 37 °C. Next, samples were centrifuged to remove the solid fat and the remaining suspension was filtered with cell strainers of first, 100 μm and afterwards 40 μm, to eliminate residual tissue parts. The obtained liquid-phase extracts were L/R labeled and stored for further experiments at − 20 °C.

MDA-MB-231 were conditioned with a cocktail consistent of 49% DMEM with Penicillin/Streptomycin, 1% Serum Fetal Bovine and 50% left or right liquid-phase extract.

### Monitoring changes in Ca^2+^ concentration and Δψ_*p*_

Cells were cultured on 30 mm glass coverslips for performing Ca^2+^ imaging. Coverslips with cells attached were mounted in a chamber and incubated at 37 °C and protected from the light for 30 min in a culture medium containing 3 μM Fluo3-AM (Invitrogen, Cat# F1242). After incubation, cells were washed two times with PBS 1× and bathed in DMEM medium supplemented with 10% fetal bovine serum, 100 U/mL of penicillin, and 100 μg/mL streptomycin for 5 min before Ca^2+^ measurements were made on an inverted Olympus FV 1000 confocal microscope (Olympus Corporation, Tokyo, Japan). Images were collected using the Fluoview FV-1000 software and an Olympus 20X lens (UPlanSApo 20X/0.75). Fluo 3 fluorescence was detected using the filter cube U-MWB2 (excitation BP 460–490 nm and emission LP 520 nm).

Still Images of cohorts of 50–100 cells were analyzed with Microsoft Excel and Image J (National Institutes of Health, USA, RRID:SCR_003070). Fluorescence data was calculated as mean/area.

To determine the time-window at which L/R treated cells acquired differences in their Ca^2+^ fluorescence curves, two experimental approaches were used. A first one, with a day-by-day stepwise assay, collecting still images of Fluo 3 every day, for 5 days with a Nikon TE300 Inverted Fluorescence Phase Contrast Microscope using ×20 magnification (Objective: PLAN FLUOR 20x/0.45), filter cube B-2A, 450–490 nm for excitation, and 500 nm dichroic and 515 nm barrier. The exposition time was 150 ms. The images were taken with a LUCA ANDOR EMCCD camera. Still Images of cohorts of 15–75 cells were analyzed with Microsoft Excel and Image J (National Institutes of Health, USA, RRID:SCR_003070). Fluorescence data of L-treated, R-treated and non-treated cells (control) was calculated as mean/area. With a second approach based on real-time experiments on a Zeiss-Axio-Observer microscope at 37 °C with 5% CO_2_, images were collected every 15 min with ×20 magnification during 3 days (since the cells did not survive longer on this system). In the stepwise approach, independent wells were each day incubated with Fluo 3, and the fluorescence values were calculated as means of still images of a 50–100 cells per field. In the real-time experiments, the fluorescent probe was first loaded and afterwards the L/R extracts were added to cells. Fluorescence data was recorded for 15–75 single cells per condition, over the 3-day-experimental time.

For Δψ_***p***_ measurements,  10^4^ MDA-MB-231 cells were plated and conditioned with L/R extracts for 5 days as described above, and then incubated for 30 min with 1 μM DiBAC_4_(3) (Bis-1,3-Dibutylbarbituric AcidTrimethine Oxonol, a fluorescent probe for membrane potential determination) (Invitrogen by Thermofisher Scientific, Cat. No. B438) at 37 °C and 5% CO_2_. Afterwards, cells were trypsinized and fluorescence was measured by flow cytometry (FACSARIA-III, BD-Biosciences®) with a BP 530/30 emission filter. Results were analyzed using FlowJo v X.0.7® software (RRID:SCR_008520).

To provoke maximum depolarization (considered as 100% depolarization), cells were first treated with 65 mM KCl for 5 min at 37 °C and afterwards incubated for 30 min with the fluorescent probe DiBAC_4_(3) as described above.

### Local breast cancer female patient mitotic index data

From a previous work of our group (Campoy et al. 2016), we counted with a database of 95 breast cancer female patients (mean age 54, range 31–86) who had previously signed an informed consent approved by the Ethics Committee of the Medical School of the National University of Cuyo, Mendoza, Argentina. The database included information of the tumor mitotic index provided by the same anatomo-pathologist. In brief, at least 10 different areas had been counted and cells in metaphase, anaphase or telophase were considered in mitosis as indicated in Ogston et al. (2003). We dichotomized the data as *low mitotic index* with a mean of up to 19 mitotic cells/area and *high mitotic index* with a mean of 20 or more mitotic cells/area.

### Statistical analyses

Differences between 2 proportions of *hyper/depolarizing* ICH were calculated as Odds Ratios (OR), with the corresponding 95% CI. To compare means and medians of fluorescent-probe concentrations, unpaired T-test was applied with Welch’s corrections when variance was not equal among L/R data. L/R ratio differences were analyzed by One sample T-test with hypothetical Right value = 1 (assigning the values of Right as reference). When more than two groups were compared, one or two-way ANOVA test were applied (with Dunnett post-test). Finally, Fisher’s exact test was used to compare categorical data. *P* values below 0.05 were considered as statistically significant.

## Results

### DNA methylation differences

#### In-silico L/R DNA methylation differences in breast tumors

**Fig. 1:**
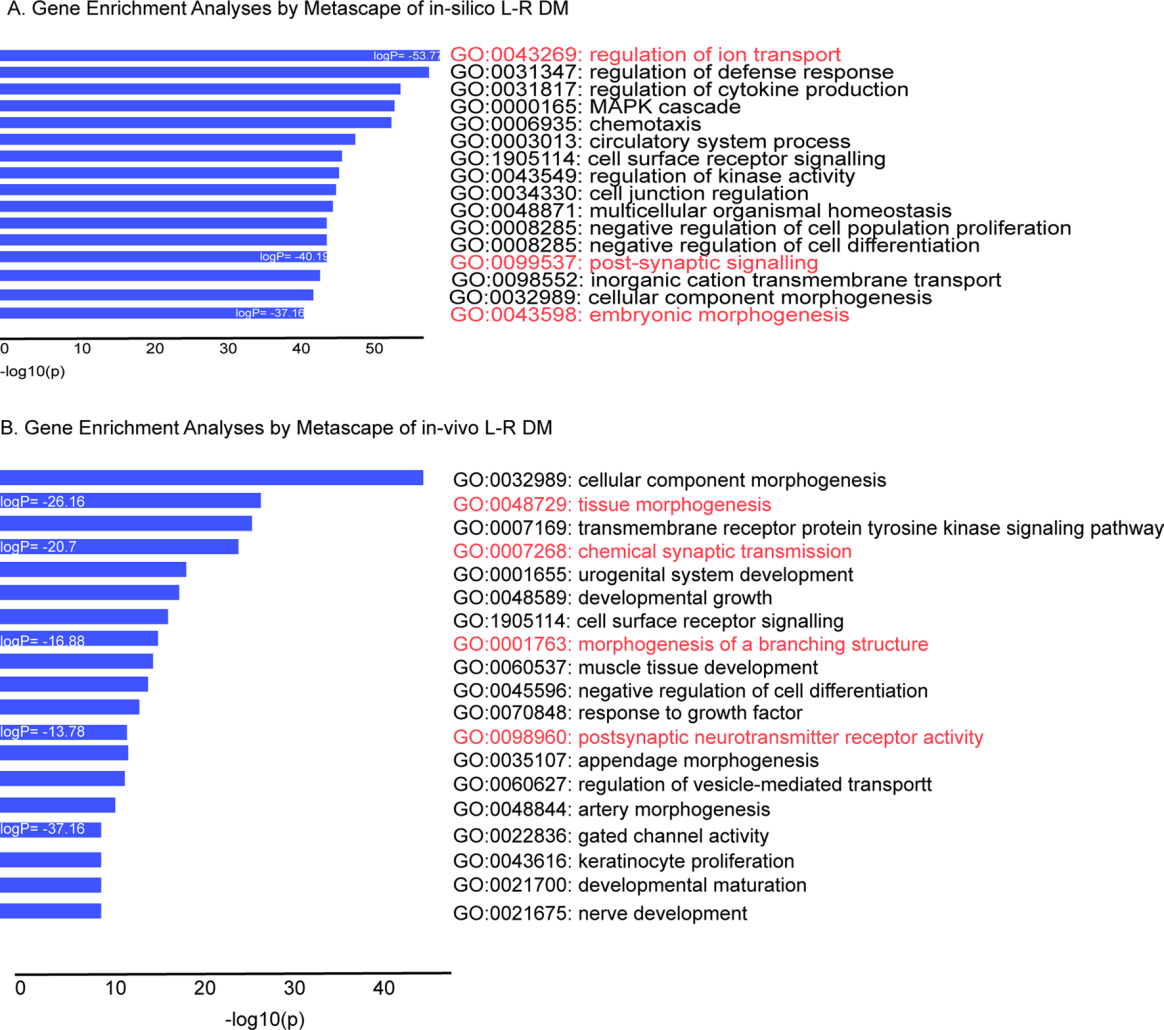
Gene enriched pathways of L/R differential methylated regions. **A** DNA regions with more than 1% DM between L–R primary breast tumors from TCGA public dataset were analyzed by Metascape for enriched GO Biological processes (p value cut-off 0.001). **B** DNA regions with more than 10% DM between 6 L/R xenograft tumors were analyzed by Metascape for enriched GO Biological processes (p value cut-off 0.001). Ion/neural and embryonic pathways are highlighted in red

The methylation profile of ~ 16,000 genomic regions were analyzed in 782 primary breast carcinomas (394 L and 388 R). We calculated the L/R DNA methylation mean for each genomic region and ranked their absolute differences (called from now on differential methylation, DM), which ranged between 10^–7^ and 5%, with a median value = 0.03%. We decided to focus on the top 2997 genes with > 1.24% difference (Additional file 2: Table S2). Gene Enrichment analyses performed on the selected genes by Metascape (https://metascape.org) revealed that the main pathways in which they were involved were related to *regulation of ion transport* (GO:0043269), *trans-synaptic signaling* (GO:0099537), and *embryonic morphogenesis* (GO:0043598) (Fig. 1A). By the tool EnrichR (https://maayanlab.cloud/Enrichr/enrich), also *embryonic digestive tract development* (GO:0048566)*, chemical synaptic transmission (GO: 0007268), calcium ion transport (GO: 0006816),* and *positive regulation of ion transport* (GO:0043270) appeared as significantly involved pathways (adjusted p values < 0.05) (data not shown).

#### In-vivo L/R DNA methylation differences in an animal model

To study whether the L/R DM was reproducible in an animal model, we generated synchronic L/R breast tumors in Nod-Scid-Gamma (NSG) immune depressed mice by inoculating the human breast cancer cell line MDA-MB-231 simultaneously in both 4th mammary glands. After generating three sequential passages (labeled as P_0_, P_1_, and P_2_), the tumors were first analyzed in a reduced number of CpG sites by Methyl-Specific-MLPA (50 CpG sites located on 40 tumor suppressor genes). The three passages showed subtle L/R differences in several genes, and we chose P_1_ as the passage with the major DM. In 15 CpG sites of 11 genes (*RASSF1A*, *ESR*, *IGSF4*, *CDH13*, *MGMT*, *TP73*, *WT1*, *MSH6*, *PAX6*, *GATA5*, and *RARB*) the L/R DM per site were from − 6.8 to 11.17%. These observations were considered only useful for choosing the best tumor passage to scale up experimentally and perform a whole genome methylation analysis.

In the light of this, DNA of the three P_1_ L/R paired xenograft tumors was selected for Reduced Restricted Bisulfite Sequencing (RRBS) assays. After establishing an arbitrary cut-off of 10% absolute DM, (see section ‘Methods’ for more details), we decided to discard inflammation-response genes, since they can increase their expression after surgical manipulation (Russell et al. 2015). Besides, after selecting one CpG site per gene (with the highest DM), 1288 genes remained (Additional file 1: Table S1), on which gene enrichment analyses were performed by Metascape. The analyses revealed *cellular* (GO:0032989) and *tissue morphogenesis* (GO:0048729), several *developmental pathways* (e.g., GO:0001655; GO:0021700; GO:0021675), *morphogenesis of a branching structures* (GO:0001763) and *chemical synaptic transmission* (GO:0007268) as the main GO biological processes (Fig. 1B). Furthermore, Genomic Region Enrichment Analyses (GREAT) (McLean et al. 2010) showed that the overall differences were mainly involved in the GO biological processes *embryonic camera-type eye development* (GO:0031076), *epidermis development* (GO:0008544), *mammary gland development* (GO:0030879), and *regulation of neuronal synaptic plasticity* (GO:0048168) (data not shown).

So very interestingly, the generated animal model revealed biological processes following what we previously had found in human in-silico data of breast tumors, indicating that L/R differences were consistently associated with *embryogenic* and *neuronal* features.

#### Focus on ion-channel genes among the in-silico and in-vivo L/R methylation differences

The role of electrochemical gradients in neurons is well known. However, an increasing amount of literature is revealing the role of electrochemical gradients in the regulation of diverse functions of *non*-neuronal cells, including morphogenesis of numerous embryonic and adult structures (Bates 2015; Harris 2021; Levin et al. 2017). Consistently, the GO term *ion transport* had appeared among the in-silico main enriched pathways. We therefore decided to search whether ion-channel genes (ICH) were included in our DM lists.

**Fig. 2:**
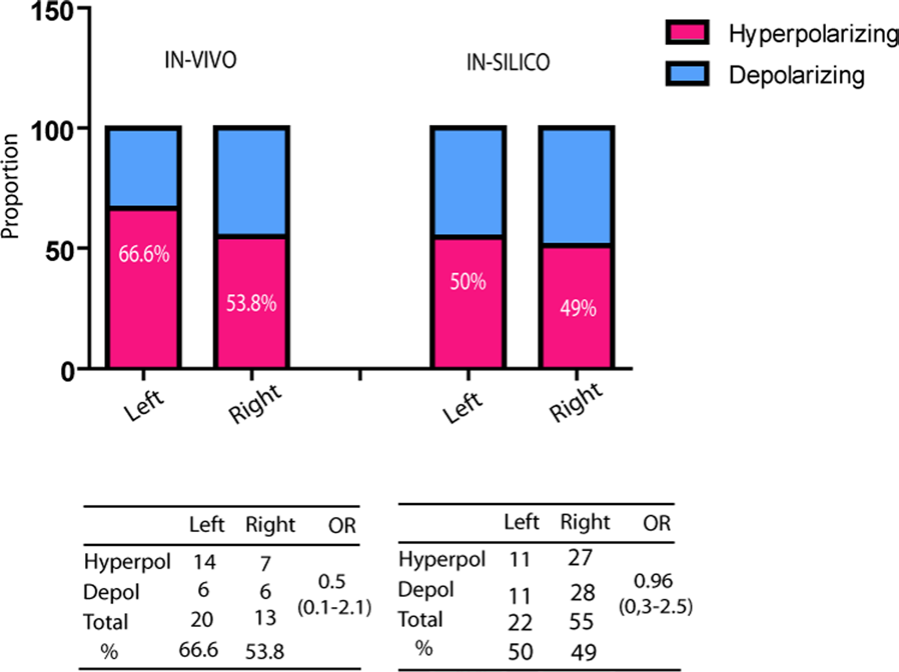
L–R comparison of methylated hyper vs depolarizing ion channels. Data from in-vivo and in-silico analyses. Tendency shows in both approaches R-sided tumors with subtle decreased proportion of methylation in hyperpolarizing channels, as compared to L ones (53.8% vs 66.6% in-vivo; 49% vs 50% in-silico)

We found 33 ICH genes matches (using as reference the Human Gene Nomenclature Committee (HGNC) ion channel list (https://www.genenames.org/data/genegroup/#!/group/177) among the in-vivo DM and 77 ICH genes in the in-silico data (Table 1). In Table 1, we have indicated for each gene an increased methylation in L-sided tumors as “more methylated left” -MML-; and decreased methylation in L as “more methylated right” -MMR-. Even though slighty, we see a tendency for decreased percentage of *hyperpolarizing* channels among the MMR genes, as compared to MML ones (53.8% vs 66.6%, OR 0.5, 95%CI 0.11–2.13 in the in-vivo experiment; 49.01% vs 50%, OR 0.96, 95%CI 0.35–2.59 in the in-silico experiment) (Fig. 2). Even though the differences did not reach statistical significance, it suggested a possible non-stochastic pattern. We reasoned that it could be possible that the L/R breast tumor differences occurred at bioelectric levels, preserving a consistent voltage change direction probably in a non-specific gene manner.

**Table 1:** L–R differential methylation of ion channels in in-vivo and in-silico studies

More methylated right		More methylated left	
Gene symbol*	Channel function	Gene symbol*	Channel function
Ion channels from in-vivo assays			
*CHRNE*	Depolarization	*ASIC2*	Depolarization
*HTR1A*	Depolarization	*CACNA1A*	Depolarization
*PIEZO2*	Depolarization	*CACNA2D2*	Depolarization
*RYR3*	Depolarization	*CATSPERD*	Depolarization
*TRPC7*	Depolarization	*PKD1L1*	Depolarization
*TRPM8*	Depolarization	*TRPM4*	Depolarization
*ANO2*	Hyperpolarization	*ANO3*	Hyperpolarization
*ATP1A3*	Hyperpolarization	*ANO5*	Hyperpolarization
*ATP6V1C2*	Hyperpolarization	*CLCN1*	Hyperpolarization
*ATP6V1H*	Hyperpolarization	*CLIC5*	Hyperpolarization
*GABBR1*	Hyperpolarization	*GABBR2*	Hyperpolarization
*KCNA7*	Hyperpolarization	*GABRA5*	Hyperpolarization
*KCNB1*	Hyperpolarization	*GABRD*	Hyperpolarization
		*GABRG1*	Hyperpolarization
		*KCNH2*	Hyperpolarization
		*KCNIP3*	Hyperpolarization
		*KCNJ18*	Hyperpolarization
		*KCNK9*	Hyperpolarization
		*KCNN1*	Hyperpolarization
		*VDAC2*	Hyperpolarization
Ion channels from in-silico assays			
*CACNA1D*	Depolarization	*CACNA2D2*	Depolarization
*CACNA1H*	Depolarization	*CACNA2D4*	Depolarization
*CACNA1I*	Depolarization	*CHRNA1*	Depolarization
*CACNB2*	Depolarization	*CNGA1*	Depolarization
*CACNG4*	Depolarization	*HCN1*	Depolarization
*CACNG6*	Depolarization	*P2RX4*	Depolarization
*CHRNA6*	Depolarization	*PKD2*	Depolarization
*CHRNB1*	Depolarization	*RYR1*	Depolarization
*CHRNB2*	Depolarization	*SCN11A*	Depolarization
*CNGA3*	Depolarization	*SCNN1A*	Depolarization
*GRIA1*	Depolarization	*TRPM3*	Depolarization
*HVCN1*	Depolarization	*CFTR*	Hyperpolarization
*LRRC8D*	Depolarization	*CLCN1*	Hyperpolarization
*LRRC8E*	Depolarization	*CLCNKB*	Hyperpolarization
*MCOLN2*	Depolarization	*KCNA1*	Hyperpolarization
*MCOLN3*	Depolarization	*KCNA5*	Hyperpolarization
*PKD2L2*	Depolarization	*KCNC3*	Hyperpolarization
*SCN3B*	Depolarization	*KCNH7*	Hyperpolarization
*SCN9A*	Depolarization	*KCNH8*	Hyperpolarization
*SCNN1G*	Depolarization	*KCNJ9*	Hyperpolarization
*TRPA1*	Depolarization	*KCNMA1*	Hyperpolarization
*TRPC2*	Depolarization	*KCNS2*	Hyperpolarization
*TRPC3*	Depolarization		
*TRPM2*	Depolarization		
*TRPM6*	Depolarization		
*TRPV3*	Depolarization		
*TRPV4*	Depolarization		
*TRPV6*	Depolarization		
*CLIC3*	Hyperpolarization		
*GABRA2*	Hyperpolarization		
*GABRG1*	Hyperpolarization		
*GABRP*	Hyperpolarization		
*GABRR1*	Hyperpolarization		
*GLRA3*	Hyperpolarization		
*GLRB*	Hyperpolarization		
*KCNA2*	Hyperpolarization		
*KCNA6*	Hyperpolarization		
*KCNA7*	Hyperpolarization		
*KCNG1*	Hyperpolarization		
*KCNH1*	Hyperpolarization		
*KCNH4*	Hyperpolarization		
*KCNH6*	Hyperpolarization		
*KCNJ15*	Hyperpolarization		
*KCNJ2*	Hyperpolarization		
*KCNJ5*	Hyperpolarization		
*KCNJ6*	Hyperpolarization		
*KCNK3*	Hyperpolarization		
*KCNK4*	Hyperpolarization		
*KCNK5*	Hyperpolarization		
*KCNN4*	Hyperpolarization		
*KCNQ3*	Hyperpolarization		
*KCNQ4*	Hyperpolarization		
*KCNS1*	Hyperpolarization		
*KCNS3*	Hyperpolarization		
*KCNT2*	Hyperpolarization		

*HUGO Gene Nomenclature Committee (HGNC)

Taken together so far, L/R epithelial carcinomas presented methylation differences in genes involved in embryogenic and neuronal processes, suggesting a pattern in ion channel genes, by decreased DNA methylation of hyperpolarizing genes on R-sided tumors. Inferring from our xenograft experiments, we could discard that these differences were original of the tissue where the tumor started (since mice-genomic interferences had been filtered), which allowed us to adventure that they were acquired during the tumor progression. To establish the environmental role in acquiring bioelectric and DNA methylation differences, we further continued with in-vitro studies.

### Bioelectric differences

#### In-vitro L/R Ca^2+^ differences in conditioned cell culture

**Fig. 3:**
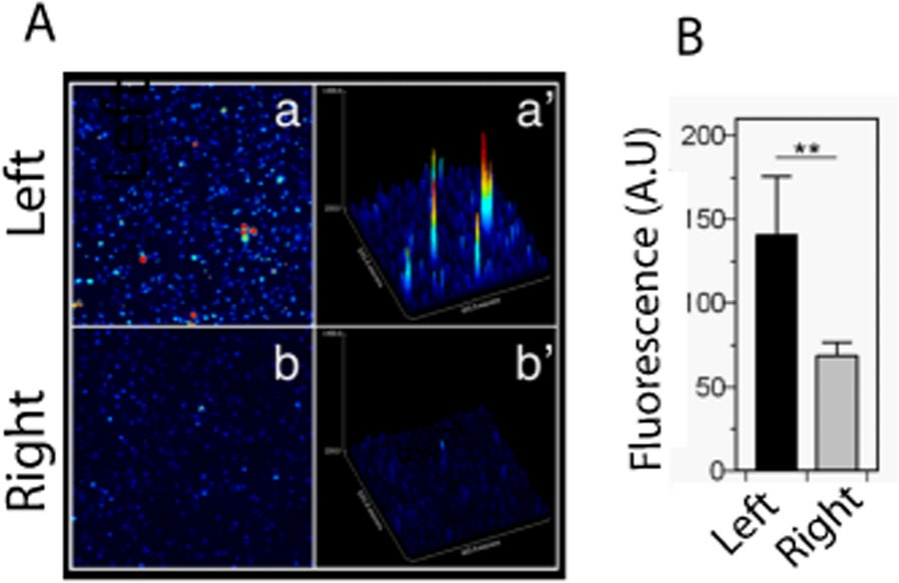
L/R Ca^2+^ concentration comparison. MDA-MB-231 cells treated with L/R normal breast extracts from donor W1 and measurement of intracellular calcium concentration with Fluo3 AM. **A** Representative calcium fluorescence in two-dimensional (a and b) and three-dimensional images-surface plot- (a′ and b′). Pseudo-color from black to red represents low to high intracellular calcium concentration, respectively. **B** Fluorescence quantification was performed on still images (×20 magnification), by calculating the fluorescence mean of 50–100 cells fields. The analysis reveals a significant increment of intracellular calcium concentration in the L-treated cells. Values represent the mean ± SEM from 3 independent experiments, 50–100 cells were analyzed per experiment (unpaired T-test with Welch’s correction, p < 0.003). *A.U* artificial units

To establish how (or if) the mammary gland microenvironments contributed to the L/R voltage differences, we set up an in-vitro model where cellular extracts of healthy L/R human mammary tissue were used to induce changes in cultured cells. From surgical reductions of healthy L/R mammary glands, we included samples from four women (median age 34) in this study. Paired L/R cellular extracts from one female donor (W1) were first used to treat MDA-MB-231 breast cancer cells for 5 days, and afterward measure Ca^2+^ concentration with a calcium fluorophore by confocal microscopy. We counted between 50 and 100 cells for each condition and confirmed that the L/R W1 extracts had a different effect on cells, showing an increased Ca^2+^ concentration in the L-treated cells (Fig. 3) (unpaired T-test with Welch’s correction, p < 0.003). With this, we confirmed that the developed in-vitro model was sensitive and reproducible to test the effect of L/R extracts on cellular electricity. Furthermore, the generated difference in Ca^2+^ concentration suggested that L-treated cells had relatively depolarized the membrane.

**Fig. 4:**
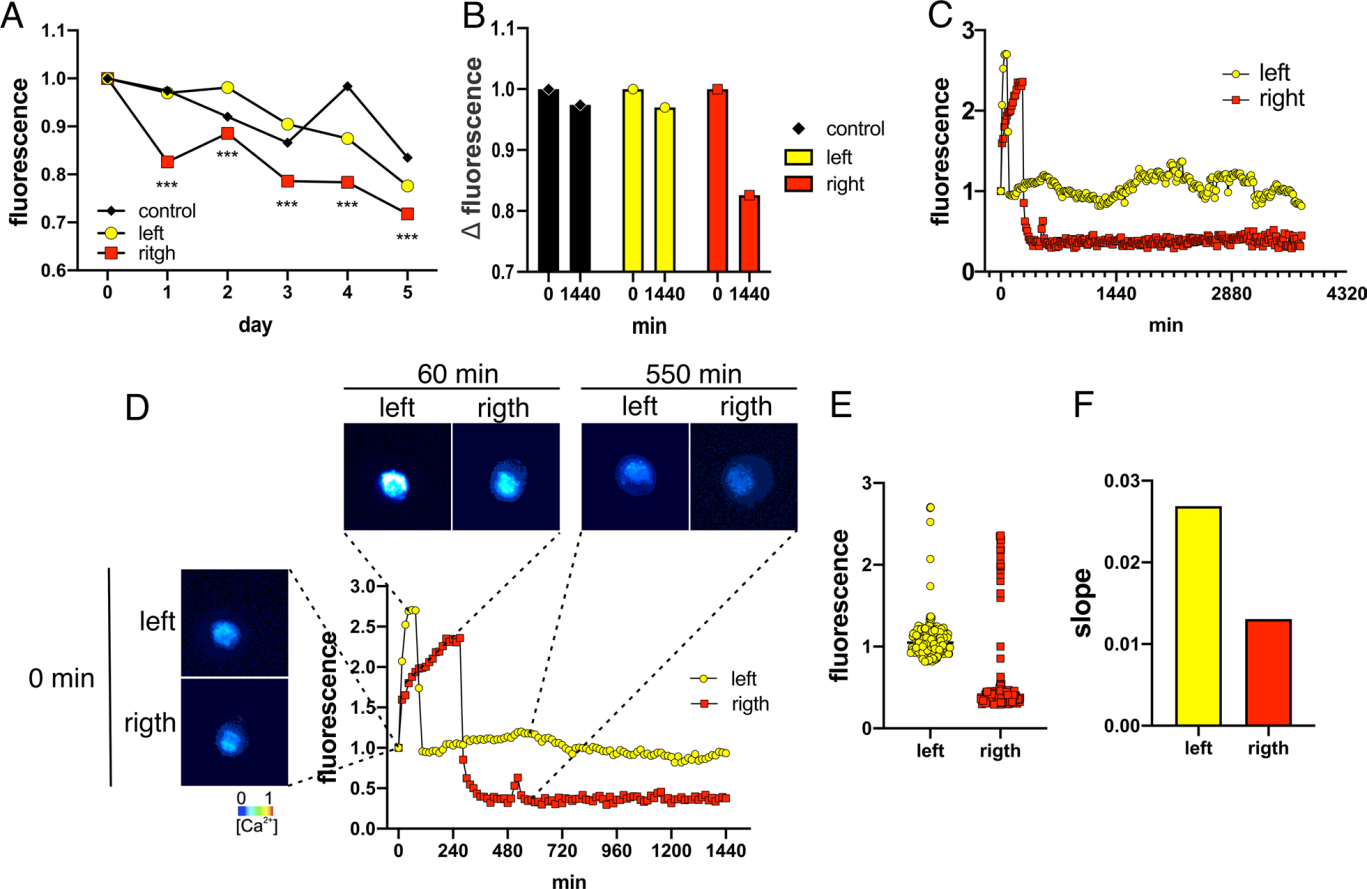
Calcium dynamics in MDA-MB231 cells treated with L- and R-extracts. **A** Mean of intracellular Fluo-3 fluorescence measured day-by-day in a Nikon TE300 Inverted Fluorescence Phase Contrast Microscope, in L, R and Control conditions. Still Images of cohorts of 15–75 cells per condition were analyzed with Microsoft Excel and Image J (National Institutes of Health, USA, RRID:SCR_003070) during 5 days. Fluorescence data was calculated as mean/area. As can be observed, in the R-treated cells intracellular Ca^2+^ concentration decreases at day 1, (as compared to controls and to L-treated cells) and this difference is maintained along the 5 days (Kruskal–Wallis test for: L/R differences: p < 0.0001;Control/R differences: p < 0.001, indicated by asterisks). While L-treated cells, despite some fluctuations during days 2–3–4, do not significantly change from the untreated cells (Kruskal–Wallis test: Control/L differences: day 1, 2, 3 and 5: ns, day 4 p < 0.01). **B** The Δ fluorescence of **A** (initial fluorescence − final fluorescence) was measured at time 0 and 1440 min (24 h). This shows how R-treated cells decreased the intracellular Ca^2+^ concentration, which is not observed in the other conditions. **C** Representative Ca^2+^ fluorescence plot in real time, of single cells in L- and R-conditions, during 3 days. **D** Representative Ca^2+^ fluorescence plot in real time and representative single cell spatiotemporal fluorescence changes in the first 1440 min (24 h). Representative pictures of different times are shown (0, 60 and 550 min). The pseudocolor from black to red represents low to high concentration, respectively. **E** Scatter plot of real-time fluorescence in L- and R-conditions shown in **C**. **F** Plots of slopes of Ca^2+^ increments during the first 360 min (6 h) shown in **C**. The L-slope is markedly greater than R-slope

To understand the underlying Ca^2+^ dynamics, we designed a stepwise approach, by which independent wells were each day incubated with Fluo3 and the fluorescence values were calculated as means of still images of 15–70 cells per field. By this, we observed that R-treated cells decreased the Ca^2+^ concentration at early times (at day 1), as compared to L-treated and to untreated control cells (Kruskal–Wallis test for: L/R differences: p < 0.0001;Control/R differences: p < 0.001). On the contrary, L-treated cells maintained a similar initial Ca^2+^ concentration and did not differ from the control cells. Even though some fluctuations occured during day 2–3–4, the L/R significant difference was maintained along the 5 days (Kruskal–Wallis test: Control/L differences: day 1, 2, 3 and 5: ns, day 4 p < 0.01) (Fig. 4A, B).

In addition, we incubated the cells with Fluo3 together with the L/R extracts and collected images with a real-time approach every 15 min during 3 days (we were not able to extend the experiment to 5 days, since cells did not survive longer in a reduced chamber and glass-surface) using a Zeiss-Axio-Observer microscope. This experiment confirmed how L- and R-treated cells show differential Ca^2+^ uptake dynamics during the first hours of treatment and maintain a differential concentration along the measured days (Fig. 4C, E). Focusing on the first 24 h (Fig. 4D), it can be observed how the Ca^2+^ dynamism slopes differ, since L-treatment provoked a rapid and strike increment in the Ca^2+^ fluorescence while R-treated cells respond slower showing a lower fluorescence increment (Fig. 4F).

To further explore this, we advanced with membrane potential analyzes.

#### In-vitro L/R $\boldsymbol{\Delta }{{\varvec{\psi}}}_{{\varvec{p}}}$ differences in conditioned cell culture

The voltage-sensitive dye specific for plasma membrane potential ($\Delta {\psi }_{p}$) Bis-(1,3-Dibutylbarbituric Acid) Trimethine Oxonol (DiBAC_4_(3)) was used to measure the effect of L/R mammary tissue extracts on MDA-MB-231. The dye (negatively charged) accumulates into depolarized cells. It has been previously established by others that this method is reliable for bioelectric studies in non-neuronal cancer cells which are known to be less polarized than normal cells (Bonzanni et al. 2020).

**Fig. 5:**
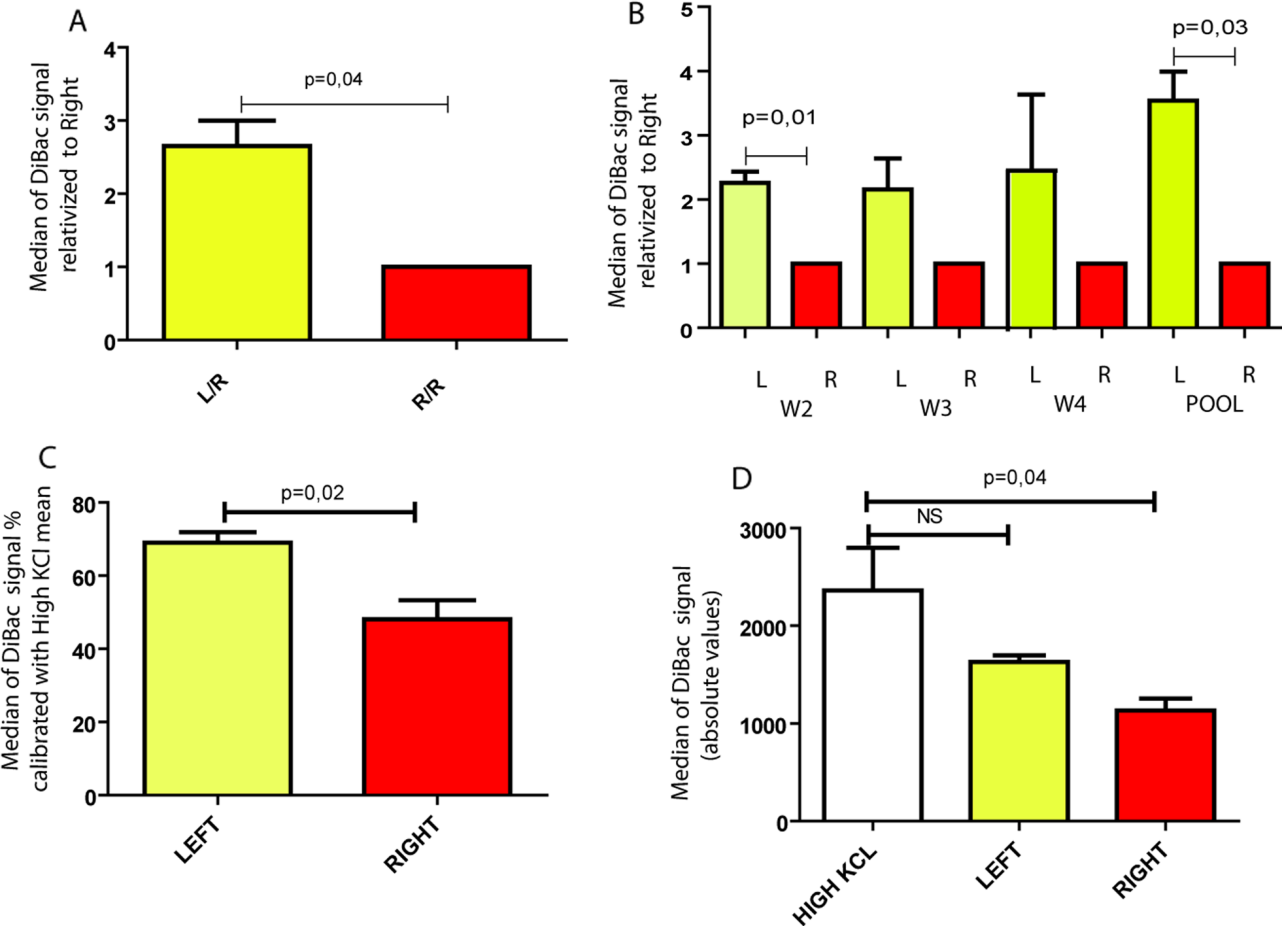
L/R membrane potential comparison in conditioned MDA-MB-231 cells. **A** The results are expressed as the L-median of DiBAC_4_(3) fluorescence relativized to R. The histogram shows the L/R and R/R ratio of the signal. The value of R/R ratio is 1. The mean value of L/R ratio is 2.65, N = 3 experimental replications (treatments with extracts W2, W3, W4; each with 2–3 technical replications). **B** Individual analyses of the effect of L/R normal breast tissue extracts on cells. The results are expressed as the L DiBAC_4_(3) fluorescence relativized to R, as explained for **A**. The three extracts present increased DiBAC_4_(3) fluorescence in L-treated cells, although not all significant. W2: p = 0.01, mean of difference 1.65; W3 and W4: L-treated NS increased tendency; pool: p = 0.03, mean of difference: 2.53. One-sample T-test with hypothetical R value = 1, for all experiments 2–3 technical replicates were performed. **C** L and R DiBAC_4_(3) fluorescence medians are expressed as percentages of the median fluorescence of high-KCl treated cells. L-treated cells present higher fluorescence as compared to the R ones (unpaired T test, p = 0.02). **D** Median fluorescence of high-KCl, L and R treated cells are compared. Only the R-treated ones differ significantly from the completely depolarized (One-way ANOVA + Dunnett post-test, p = 0.0037). Raw data are available in Additional file 3: Table S3

The L/R extracts of three female donors (W2, W3, W4) were used to perform treatment replications. After 5 days of treatment, the DiBAC_4_(3) signal was measured by flow-cytometry (Raw data available in Additional file 3: Table S3). Interestingly, the L-treated cells displayed an increment in the fluorescence signal, indicating a less polarized state, in line with our previous assumption (L/R-fluorescence ratio, One-sample T-test with hypothetical R value = 1, p = 0.04, Fig. 5A). This observation was consistent for each extract, although not all reached the statistical significance (W2: p = 0.01, mean of difference 1.65; W3: p = 0.25 and W4: p = 0.41, 2–3 technical replicates, Fig. 5B). When we mixed the L and R extracts in a pool and compared the effect, the generated difference was statistically significant (L/R-fluorescence ratio, One-sample T-test with hypothetical R value = 1, p = 0.03, mean of difference: 2.53, three technical replications, Fig. 5B). With this, we confirmed that: (i) the extracts had a differential bioelectric effect on the treated cells, (ii) the effect was independent of the donor, and (iii) the L-extracts generated a *depolarized* state as compared to R-extracts. Having confirmed this, we chose the pooled extracts for further studies to avoid possible donor-specific bias.

Our following aim was to establish the magnitude of the L/R $\Delta {\psi }_{p}$ differences. For this, to normalize the potentials to the maximum possible depolarized state (which we established as a 100% depolarization reference value), we treated cells with a depolarizing agent (65 mM KCl, as suggested by Bonzanni et al. to depolarize MDA-MB-231 (Bonzanni et al. 2020). When expressing the DiBAC_4_(3) results as a percentage of the values of completely depolarized cells, we found that L-treated cells showed 69% (95% CI 56.67–81.35) vs R-treated 48% (95% CI 25.72–70.42), difference which was statistically significant (Unpaired T test, p = 0.02, Fig. 5C, Raw data available in Additional file 3: Table S3). Interestingly, however, no statistical difference was observed between L-treated cells and the KCl-treated ones, while R-treated cells did differ significantly (One-way ANOVA + Dunnett post-test, p = 0.0037, Fig. 5D). So we could conclude by this that the L-treated cells reached a similar depolarization as the maximum depolarized cells.

### Epigenetic enzymes differences

#### In-silico L/R methyltransferase expression differences

**Fig. 6:**
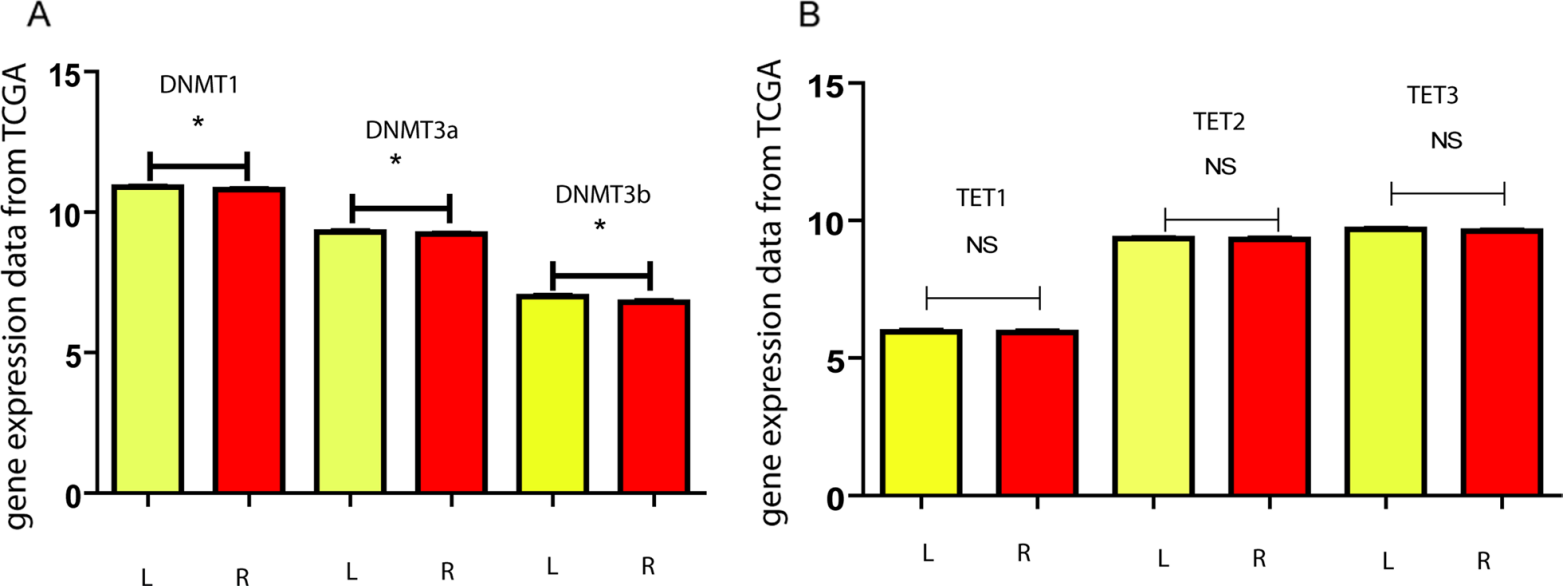
L–R epigenetic modulators expression comparison. **A** Gene expression data from 1168 TCGA L–R breast tumors for DNMTs and 1095 for TETs. *DNMT*s present significant increased expression on L-sided tumors, as compared to the R ones. This is not accompanied by the *TET* genes (**B**), which do not differ in their expression regarding the side

It has been well documented that epigenetics has a role in the adaptive regulation of gene expression. Specifically in neurons, in the dynamic expression of ion channels it has been reported that enzymes involved in DNA cytosine methylation have a crucial participation (Meadows et al. 2016). The process is catalyzed by DNA methyltransferases (DNMTs) and most commonly occurs at cytosines followed by a guanine, called CpG sites. DNMT3A is a de novo DNMT that methylates cytosines on unmethylated CpG sites, while DNMT1 is a maintenance DNMT that methylates cytosines on an unmethylated CpG with a methylated opposite strand. The inverse *de*-methylation process is regulated by the ten-eleven translocation (TET1, 2, and 3) family enzymes which oxidate the 5-methylcytosine to 5-hydroxymethylcytosine. We wondered whether these enzymes were differentially expressed in L/R mammary tumors. Previous work by others have reported that the expression of DNMTs is associated with the total DNMT activity (Maugeri et al. 2019; Tolg et al. 2011). From the TCGA-dataset of the Xena Functional Genomics Explorer (https://xena.ucsc.edu/), 1168 primary breast tumors (584 L and 584 R) were analyzed for *DNMT*s gene expression. L breast tumors presented increased expression of the 3 *DNMT* types (non-normally distributed data, Welch unpaired T-test, *DNMT1*: p = 0.01; *DMT3a*: p = 0.04; *DMT3b*: p = 0.001) (Fig. 6A). Instead, the demethylating enzymes *TET1*, *2* and *3* did not present any difference associated with laterality in 1095 primary breast tumors (571 L and 524 R) (Fig. 6B, non-normally distributed data, Welch unpaired T-test, p > 0.05). As control, normal tissue was analyzed where none of the studied enzymes presented L/R differences. When comparing normal vs tumoral, all the enzymes had altered expression in tumors. The 3 *DNMT*s presented significantly increased expression (Unpaired T test, p < 0.0001), *TET 1* and *2* decreased expression (Unpaired T test, p < 0.001), and *TET3* increased expression (Unpaired T test, p < 0.001), as compared to their side-respective normal tissue.

The observations suggest that, independently of the TET enzymes, the DNMTs are increased on the L-sided tumors, when compared to the R-ones. When deepening on the TET/DNMT relationship, it has been recently shown that TETs do compete with DNMTs in promoters of genes associated primarily to development and morphogenesis (Zhou et al. 2018). TETs act maintaining a hypomethylated state in these promoters, only in the absence of DNMTs. Based on these recent findings, one could ask whether increased methylation of hyperpolarizing ICH in L-tumors is due to enhanced DNMT activities, or/and if in R-tumors, with less DNMT activity, the TET enzymes are more actively demethylating the hyperpolarizing ICH.

### Proliferation differences

#### In-silico L/R differences in KI67 expression

**Fig. 7:**
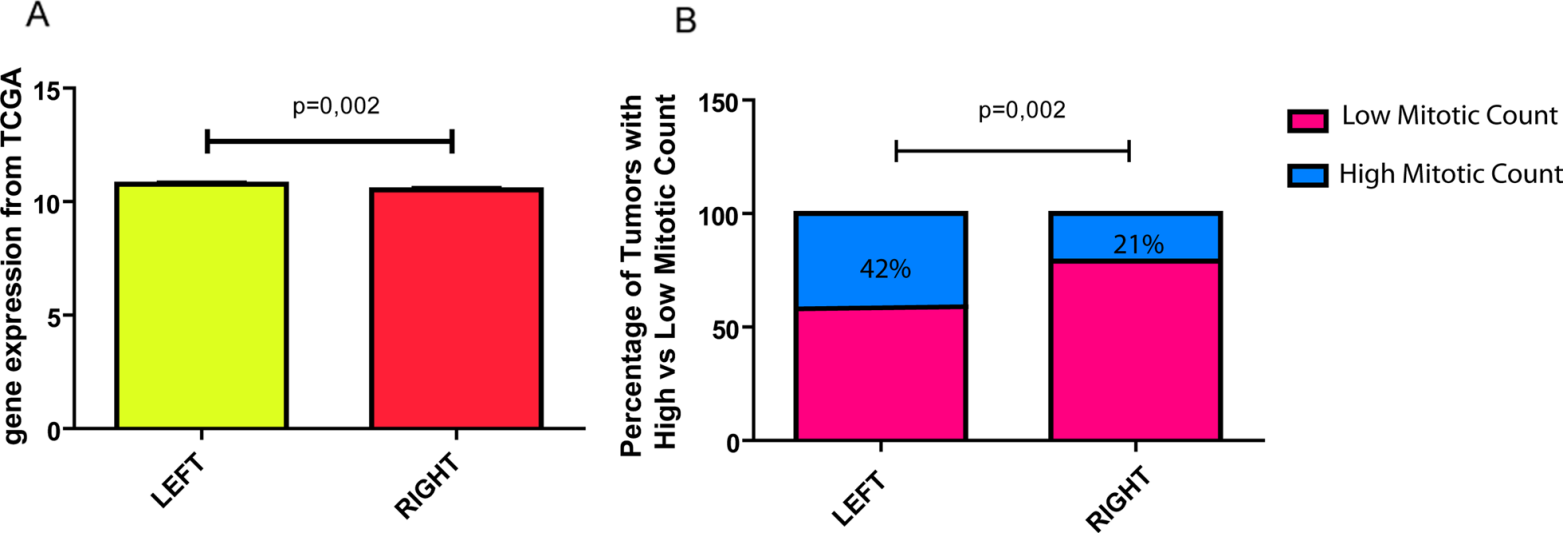
L–R proliferation rate comparison by *KI67* and mitotic index. **A** Comparison of L–R *KI67* expression in 1060 primary breast tumors from TCGA. L expression is subtle but significantly increased (10.76 ± 0.05259 N = 571) as compared to R (10.52 ± 0.05836 N = 524) (Unpaired T-test, p = 0.002). **B** Proportion of 95 L–R IDC breast tumors from local female patients with high and low mitotic count (Fisher’s exact Test, p = 0.002)

A cell that needs to divide enters the cell cycle, and the regulation of the progression from one phase to the next one has been proposed to be coupled to environmental conditions so that this occurs only when it is necessary (Inzé and Veylder 2006). It is also known that the activity and expression of ion channels change during the cell cycle, and that Ca^2+^ concentration increases at the 3 cell cycle checkpoints and the membrane depolarizes between G_2_ and Mitosis, as reviewed by Rosendo-Pineda et al. (2020). We decided therefore to analyze whether the L/R tumors presented proliferation differences. The protein KI67 is widely used as a proliferation marker in different types of tumors. In-silico databases contain RNAseq values of *KI67*, obtained by Illumina HiSeq RNA Sequencing. We searched in breast in-silico datasets the expression of *KI67* and matched it with the tumoral laterality information. Of 1060 primary breast tumors of the TCGA breast cancer dataset, we found a significant increment of *KI67* expression in L-sided tumors (L: 10.76 ± 0.05259; R: 10.52 ± 0.05836; Unpaired T-test, p = 0.002, Fig. 7A). Normal L/R breast tissue did not present differences in *KI67* expression (Unpaired T-test, p > 0.5). Both observations suggest a subtle increment of proliferation in L-sided tumors, as compared to the R-sided. This is consistent with our bioelectric findings since a depolarized state is necessary for cells to enter in mitosis.

#### In-vivo L/R mitotic index differences in female patient breast tumors

From our previous publication (Campoy et al. 2016) we counted with a database of clinic-pathological information of 95 local female patients with breast cancer (mean age 54, range 31–86). Revising the data, we analyzed if side correlated with the tumoral mitotic count. To establish the mitotic index, 10 different areas had been counted and cells in metaphase, anaphase or telophase were considered in mitosis. We classified the tumors as *low mitotic index* with a mean of up to 19 mitotic cells/area and *high mitotic index* with a mean of 20 or more mitotic cells/area. In concordance with in-silico observations, we found significantly more tumors with high mitotic index on the L side (Fisher’s exact Test, p = 0.002, Fig. 7B).

## Discussion

Bioelectric fields are produced naturally in all living tissue. Not only excitable nerve and muscle cells, but all cell-collectives that are organized in a functional network generate bioelectric signals to communicate among each other. Long before neurons existed, evolution exploited bioelectric networks to regulate morphogenesis and behavior (Fields et al. 2020; Martinez-Corral et al. 2019). When multicellular organisms appeared, the same efficient mechanism has been conserved for long-distance communication at different levels of the whole body. Bioelectric gradients are involved in embryogenic processes, such as eye development (Pai et al. 2012b), brain shape (Pai et al. 2015), antero-posterior and L/R axes (Levin et al. 2002), and the control of appendage size and shape (Belus et al. 2018; Perathoner et al. 2014; Lanni et al. 2019; Dahal et al. 2012). Endogenous bioelectric properties are seen to be critical due to numerous channelopathies in human and model systems, and there is an increasing realization that ion channels can also be oncogenes (reviewed in Lanni et al. 2019; Chernet and Levin 2013; Srivastava et al. 2021; Prevarskaya et al. 2018; Becchetti 2011; Rao et al. 2015; Fraser et al. 2014). In 1938, Burr et al. showed that tumorigenic processes in the mammary glands of mice were associated with disrupted bioelectric patterns in the chest (Burr et al. 1938). Since the last decades, when the tools to study bioelectricity increased significantly, many others have associated cancer with bioelectric alterations (reviewed in Moore et al. 2017) and discovered that the tumor microenvironment impacts on the bioelectric tumor pattern (Chernet and Levin 2014).

Epigenetics is also a key player in the interaction between cells and microenvironment. A rapid gene-expression shift is many times required to respond on time to the variable environment. We propose here a connection between epigenetics, environment, and bioelectric changes that the tumor cell senses, uses, and copes-with to shape a survival strategy.

In this work we have identified differences in methylation profiles and epigenetic regulators associated with distinct microenvironments (L/R), in addition to different bioelectric states and proliferation markers. We have found that L tumors present an increased expression of DNA methylation enzymes, an increased proportion of methylated hyperpolarizing ICH genes, a more depolarized membrane potential, and an increment in proliferation markers or mitotic index. These results can complement related observations of other biological and medical fields. For example, in the clinic it is well known that breast cancer has a slightly lower incidence on right sides (Roychoudhuri et al. 2006). And others have explored that hyperpolarization decreases tumor incidence (Sundelacruz et al. 2009; Levin 2012; Chernet and Levin 2014). Our results can connect both descriptions, by proposing that the more polarized state of R-sided tumors could explain the lower tumor incidence.

How this interplay between methylation, ion channels, voltage changes and proliferation occurs, in which order they are related or whether one is causative of the other are open questions for next studies. Are the methylation profiles responsible for the bioelectric differences? We did not find a strong inverse correlation between methylation and expression of the involved ICH genes in TCGA. The expression profiles of the ion channels which were found methylated did not reveal laterality differences in in-silico data. However, we think that this is explainable by the fact that the bioelectric differences are not gene-specific. So, probably it is not possible to establish a fixed panel of ion channel genes to study L/R differences. It is also possible to think on an inverse relation between bioelectricity and DNA methylation, where the epigenetic profiles are not causative but instead are a consequence of the bioelectric alterations, as has been proposed previously by others in neurons (Cortés-Mendoza et al. 2013; Penas and Navarro 2018) and development (Tseng and Levin 2012). In any case, it is worth to state that the transcriptional profiling undertaken here is meant to characterize one important and tractable input into bioelectrical differences, however not claimed to be the only source of asymmetry. Future work will explore other possible inputs as well, such as physiological gating dynamics.

Our in-vitro model has shown to be a reliable experimental tool to electrochemically transdifferentiate cells with L/R extracts (even though not sensitive enough to reach significant gene expression differences). Although it is generally accepted that experiments in culture do not recapitulate the complexity of the cellular surroundings, our model produced repeatable and consistent bioelectric results in concordance with what was observed in-silico and in animals. This encourages to postulated it as an efficient study tool for this purpose. Again, many questions remain. What components of the L/R extracts are producing different polarization in cultured cells? Morphogens? Small molecules? Neurotransmitters? Ions?

## Conclusion

If further studies establish that general tumors on bilateral organs differ in their membrane potential, it could open new candidate therapeutic options by, for example, designing cocktails of channel openers/blockers, which are widely used in the clinic (Levin et al. 2019). The promising perspective is that, as proposed in Levin (2019), the interference with (or restoration of) bioelectric communication among tumor cells should be able to suppress carcinogenesis. Our work has opened new focuses based on L/R epigenetic and bioelectric differences in breast cancer, which could serve as prove of principle for other bilateral cancers like kidney, lung, testis, ovary and brain.

## Supplementary Information

**Additional file 1: Table S1.** Raw of 1288 differentialy methylated genes in Left and Right xenograft tumors.

**Additional file 2: Table S2:** Raw data of 2997 differentialy methylated genes from Left and Right in-silico breast tumors.

**Additional file 3: Table S3.** Raw data of DiBAC fluorescence measured by flow cytometry in Left and Right extract-treated cells.

## Data Availability

The datasets supporting the conclusions of this article are included within the article and are available in the cBioportal of Cancer Genomics (Cerami et al. 2012; Gao et al. 2013) (http://www.cbioportal.org/) and Xena Functional Genomics explorer (http://xena.ucsc.edu/, RRID:SCR_018938).

## Abbreviations

DMG: Differentially methylated genes
DNMTs: DNA methyltransferases
ICH: Ion channel
L: Left
MS-MLPA: Methylation Specific-Multiplex Ligation-Dependent Probe Amplification
NSG: Nod Scid Gamma
$\Delta {\psi }_{p}$: Plasma membrane potential
R: Right
RRBS: Reduced Restricted Bisulfite Sequencing
TCGA: The Cancer Genome Atlas
TET: Ten-eleven translocation
