# Correction to: When left does not seem right: epigenetic and bioelectric differences between left and right‑sided breast cancer

**DOI:** 10.1186/s10020-022-00457-w

**Published:** 2022-02-28

**Authors:** Sofia Masuelli, Sebastian Real, Emanuel Campoy, Maria Teresita Branham, Diego Matias Marzese, Matthew Salomon, Gerardo De Blas, Rodolfo Arias, Michael Levin, Maria Roque

**Affiliations:** 1 National Council of Scientific and Technological Research (IHEM-CONICET), PC:5500 Mendoza, Argentina; 2 Medical School, National University of Cuyo, PC:5500 Mendoza, Argentina; 3 Medical School, Mendoza University, PC:5500 Mendoza, Argentina; 4 Cancer Epigenetics Laboratory, Health Research Institute of the Balearic Islands (IdISBa), Palma, Spain; 5 Department of Medicine, Keck School of Medicine, University of Southern California, Los Angeles, CA 90007 USA; 6 Allen Discovery Center at Tufts University, Medford, MA 02115 USA; 7 Exact Science Faculty, National University of Cuyo, PC:5500 Mendoza, Argentina

## Correction to: Molecular Medicine (2022) 28:15 https://doi.org/10.1186/s10020-022-00440-5

Following publication of the original article (Masuelli et al. 2022), we have been informed that first name and last name have been swapped for 1st, 2nd, 3rd, 6th, 8th, 9th and 10th author.

The author group has been updated above and the original article has been corrected.
